# 

**Published:** 1893-11

**Authors:** 


### CONTENTS—NOVEMBER, 1893.—VOL. IX., NO. 5.

ORIGINAL COMMUNICATIONS—

Tuberculosis and The French Congress for its Study. R. H. L. Bibb, M. D., Saltillo, Mexico . . . . . . . . . . . . . . . . . . . . 199
Newspapers vs. The Doctors. Henry Ryder-Taylor, San Antonio . 206
A Mexican Remedy for Dysentery. R. T. Knox, M. D., Gonzales . 211
Medical Ethics. L. A. Grizzard, M. D., Abilene . . . . . . . . . . 214
Compound Fracture of the Lower Extremity. R. Menger, M. D., San Antonio . . . . . . . . . . . . . . . . . . . . . . . . . . . 216
Tongue Traction as a Respiratory Stimulant in Asphyxia, with Report of Cases. T. W. Shearer, M. D., Wallisville . . . . . . . . . . . 219

ABSTRACTS OF CURRENT MEDICAL LITERATURE—

Department of Surgery. Edited vy Prof. J. S. Thompson, M. D., Galveeton . . . . . . . . . . . . . . . . . . . . . . . . . . 220

CLINICAL REPORTS—

Puerperal Eclampsia. Robert Morris, Interne . . . . . . . . . . 223

CORRESPONDENCE—

Letter from Dr. W. A. Hammond . . . . . . . . . . . . . . . . . 225
Diagnosis Wanted. Dr. S. H. Barham, Caledonia . . . . . . . . . 226
Congenital Anchylosis of Fingers. Dr. J. P. Coffey, Plano . . . . . 227

EDITORIAL—

What a Fish a Erog Is . . . . . . . . . . . . . . . . . . . . . . 229
Mexican Sanitation; Sanitation on the Rio Grande . . . . . . . . 231
No Confederates Need Apply . . . . . . . . . . . . . . . . . . . 232
Dr. Hammond's Letter . . . . . . . . . . . . . . . . . . . . . . 233
Newspapers and the Doctors . . . . . . . . . . . . . . . . . . . 233
Amick's "Press Dispatches" . . . . . . . . . . . . . . . . . . . 234

MEDICAL NEWS AND MISCELLANY—

Items too numerous to even indicate in the limited space allotted; all interesting. Read them . . . . . . . . . . . . . . . . . . . 235
BOOK NOTICES . . . . . . . . . . . . . . . . . . . . . . . . . 245
PUBLISTHERS' NOTES . . . . . . . . . . . . . . . . . . . . . . 248

